# Arbuscular Mycorrhizal Fungi Induce Tolerance to Salinity Stress in Taro Plantlets (*Colocasia esculenta* L. Schott) during Acclimatization

**DOI:** 10.3390/plants11131780

**Published:** 2022-07-05

**Authors:** Obdulia Baltazar-Bernal, José Luis Spinoso-Castillo, Eucario Mancilla-Álvarez, Jericó Jabín Bello-Bello

**Affiliations:** 1 Colegio de Postgraduados Campus Córdoba, Km. 348 Carretera Federal Córdoba-Veracruz, Veracruz 94953, Mexico; obduliabb@colpos.mx (O.B.-B.); jlspinoso@gmail.com (J.L.S.-C.); euca_man90@hotmail.com (E.M.-Á.); 2 CONACYT—Colegio de Postgraduados Campus Córdoba, Km. 348 Carretera Federal Córdoba-Veracruz, Veracruz 94953, Mexico

**Keywords:** ex vitro survival, *Glomus intraradices*, mycorrhizal, colonization

## Abstract

Soil salinity is a problem that affects soil fertility and threatens agri-food crop production worldwide. Biotechnology, through plant micropropagation and the use of biofertilizers such as arbuscular mycorrhizal fungi (AMF), is an alternative to increase productivity and induce tolerance to salinity stress in different crops. This study aimed to evaluate the effect of different doses of the fungus *Glomus intraradices* on the ex vitro development of taro (*Colocasia esculenta* L. Schott cv. Criolla) plantlets under salinity stress during the acclimatization stage. In vitro-obtained *C. esculenta* plantlets were inoculated at different doses (0, 100, and 200 spores per plantlet) of *G. intraradices* during acclimatization. At 60 d of acclimatization in the greenhouse, plantlets were exposed to 100 mM NaCl salinity stress for 10 d. After the stress period, plantlet development, colonization percentage, and biomass were evaluated. In addition, the content of chlorophyll, carotenoids, proteins, proline, glycine-betaine, soluble phenols, and antioxidant capacity were quantified. The results showed differences in the developmental, physiological, and biochemical variables evaluated; however, no changes in total protein content were observed. Spore colonization showed that the symbiotic association has positive effects on the development of plantlets with or without salinity stress. This symbiotic interaction contributes to salinity stress tolerance in *C. esculenta* plantlets. The early application of AMF in in vitro-obtained taro plantlets is an alternative to increase or maintain the productivity of this crop in saline soils.

## 1. Introduction

Taro (*Colocasia esculenta* L. Schott), a member of the plant family Araceae, is cultivated due to its importance in the food industry as a result of its high content of starch, proteins, vitamins, polysaccharides, and various trace elements [1,2]. It is also sought by the pharmaceutical industry for its secondary metabolites with antitumor, antimetastatic, antioxidant, and anti-inflammatory properties [3], and by the biorefinery industry for use in bioethanol production [4]. The uses and benefits of *C. esculenta* make it a genetic resource with high commercial value. However, taro cultivation faces great challenges in terms of productivity due to little renewal of certified plant material and the abiotic stress caused by an excess accumulation of salts in the soil. Salinity affects soil fertility with effects on low crop yields [5]. Soil salinity, both of natural origin (primary salinization) and as a consequence of anthropogenic activities (secondary salinity), is a threat to agriculture and a constraint on agri-food crop production. The main factors contributing to the increase in salinity are low precipitation, high surface evaporation, use of wastewater for irrigation, groundwater depletion, and poor agricultural practices caused by the excessive use of chemical fertilizers such as carbonates, sulfates, sodium nitrates and chlorides, potassium, and magnesium, among others [6,7], coupled with a lack of leaching practices.

Plant and microbial biotechnology offer alternatives to increase crop productivity and mitigate the effects of abiotic stress on plants. One option is micropropagation using plant tissue culture (PTC) techniques to obtain certified plants with high genetic and phytosanitary quality [8], while another is the use of microorganisms such as mycorrhizae that can establish symbiotic associations with plants to stimulate greater development and increase tolerance to different types of abiotic stress [9]. Acclimatization is the final stage of micropropagation and consists of the gradual transfer of in vitro-grown plantlets to ex vitro conditions under a greenhouse [10]. In addition, plantlet acclimatization is a process that allows early inoculation of microorganisms prior to transplanting in the field for seedbed establishment. Mycorrhizae play an important role in the development and survival of their constituent species (plants and fungi) [11,12]. Arbuscular mycorrhizal fungi (AMF) are obligate biotrophs that establish endosymbiotic associations with the roots of vascular plants [13,14]. AMF enhance mineral nutrient uptake in the host plant [13,15], have positive effects on photosynthetic activity [16], decrease disease invasion [17], and confer tolerance to abiotic stresses caused by abrupt temperature changes [18], heavy metals [19], and drought and salinity stress. The positive effects of AMF on plants are the production of antioxidants and polyamines, osmotic adjustment, improved water transport in plants exposed to salinity, and maintenance of an ionic balance in the Na^+^/K^+^ ratio through the external mycelium to enhance plant development and survival under salt stress [20,21,22,23]. The use of mycorrhizae to improve salinity tolerance in plants has been evaluated in *Rhizophagus irregularis* in swamp she-oak (*Casuarina glauca*) [24], *Funneliformis mosseae* in Chinese honey locust (*Gleditsia sinensis*) [25], *Glomus mosseae* in wheat (*Triticum aestivum* L.) [26], and *Rhizophagus irregularis* in maize (*Zea mays* L.) [27]. This study aimed to evaluate the effect of different doses of *Glomus intraradices* spores during salinity stress induced during ex vitro development of *C. esculenta* plantlets at the acclimatization stage.

## 2. Results and Discussion

### 2.1. Colonization Percentage, Plantlet Development, and Biomass

**Figure 1:**
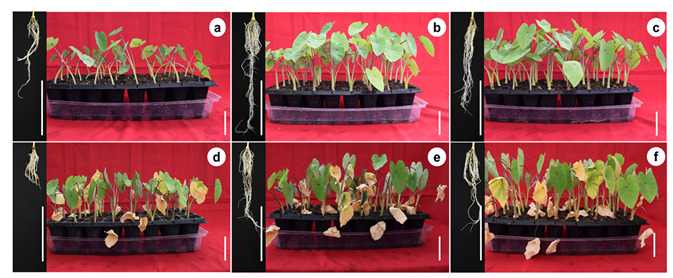
Effect of sodium chloride (NaCl) and arbuscular mycorrhizal fungi (*Glomus intraradices*) on ex vitro development of taro (*Colocasia esculenta* L. Schott) during acclimatization stage. (**a**) Non-mycorrhizal plantlets, (**b**) 100 spores per plantlet, (**c**) 200 spores per plantlet, (**d**) non-mycorrhizal plantlets + 100 mM NaCl, (**e**) 100 spores per plantlet + 100 mM NaCl, and (**f**) 200 spores per plantlet + 100 mM NaCl. White bars = 5 cm.

The different AMF application doses and induced salinity stress had an effect on the colonization percentage and developmental and biomass variables evaluated (Table 1). Regarding colonization, the highest colonization percentages were observed in plantlets inoculated with mycorrhizae without salinity stress conditions, followed by plantlets with mycorrhizae without salinity stress conditions, followed by plantlets with mycorrhizae + NaCl, whereas the lowest colonization percentage was observed in plantlets without mycorrhizae. The largest plantlets were observed at doses of 100 and 200 spores per plant without salinity stress application, whereas the smallest plantlets were obtained under salinity stress without mycorrhizae. For the number of leaves per plantlet, the highest number of leaves was observed in the mycorrhizal treatments, whereas the lowest number of leaves per plantlet was observed in the treatments without mycorrhizae. The highest percentage of senescent leaves was found in plantlets under salinity stress and without mycorrhizae, whereas the lowest percentage of senescent leaves was observed in the treatments without salinity stress, with or without mycorrhizae. For the variable number of roots, the highest number of roots was found at doses of 100 and 200 spores per plantlet without salinity stress, while the lowest number of roots was observed in the treatment without mycorrhizae and without salinity stress, and in the treatment with salinity stress without mycorrhizae. On the other hand, the greatest root length was observed at doses of 100 and 200 spores per plantlet, whereas the smallest roots were observed under salinity stress and without mycorrhizae (Figure 1). Regarding the biomass variables, the highest fresh weight was observed at doses of 100 and 200 spores with salinity stress, whereas the lowest fresh weight was observed in the treatment without mycorrhizae and without salinity stress, and in the treatment with salinity stress without mycorrhizae. The highest dry weight was observed in all mycorrhizal treatments, with or without salinity stress, whereas the lowest dry weight content was observed in the treatment without mycorrhizae and without salinity stress. For the dry matter (DM) percentage, the highest percentage was observed at doses of 100 and 200 spores per plantlet under salinity stress, whereas the lowest percentage was observed in the treatment without mycorrhizae and without salinity stress.

The results obtained in this study demonstrate the effect of AMF with or without salinity stress on colonization percentage and plantlet development and biomass variables. The AMF doses evaluated had a positive effect on plantlet development and tolerance to salinity stress. This effect could be due to the fact that mycorrhizal colonization percentages allow an adequate symbiotic interaction that could favor some physiological processes for the plant such as photosynthetic efficiency and water and nutrient uptake, keeping a high K^+^/Na^+^ ratio and higher osmoprotectants and enzyme activities [28].

Increased development and biomass in plants inoculated with mycorrhizae have been reported in melon (*Cucumis melo*) [29], watermelon (*Citrullus lanatus* L. cv. Qilin) [17] and sugarcane (*Saccharum* spp. Hybrids) [30]. Meddich et al. [29] observed in *C. melo* that when using a mycorrhizal consortium with *Glomus* sp. at a dose of 44 spores per plant, shoot and root biomass increased significantly. Wu et al. [17] observed in *C. lanatus* that colonization with mycorrhizae at a dose of 300 spores per plant promoted greater biomass accumulation. Sales et al. [30] in *Saccharum* spp. obtained an increase in yield when inoculating with native AMF at a dose of 260 spores per plant. In this study, mycorrhizal treatments with or without salinity stress increased plantlet length, number of leaves, number of roots, root length, fresh weight, and dry weight compared to treatments without mycorrhizae. On the other hand, plantlets that were not inoculated with mycorrhizae had a lower rate of development; however, none of the plantlets died at a dose of 100 mM NaCl. The zero-mortality rate in plantlets was probably due to Na^+^ accumulation in microcorm tissues; however, leaf senescence was observed in NaCl-treated plantlets. In our study, senescence in NaCl treatments could be due to older leaves showing the effects of salinity stress toxicity first, probably because they have transpired longer and therefore accumulated more Na^+^ in their tissues during the stress period causing leaf death. Lloyd et al. [31] found that *C. esculenta* was able to continue to grow and exclude sodium from transpiring leaves with 100 and 200 mM NaCl. In addition, an increase in senescent leaves and marginal leaf chlorosis were observed in salt treatments. 

In crops such as licorice (*Glycyrrhiza glabra*) [32], wheat (*Triticum aestivum*) [33], pistachio (*Pistacia vera* L. cv. Ohadi) [34], Saudi pearl millet (*Pennisetum spicatum*) [35], and maize (*Zea mays* L.) [27], an increase in the developmental and biomass variables of plants inoculated with mycorrhizae under salinity stress has been observed. The effects of mycorrhizae on development depend on the type and dose of AMF in relation to the host plant species. Amanifar et al. [32] in *G. glabra* observed that by using *Funneliformis mosseae* at a dose of 840 spores per plant, root length increased significantly under salinity stress for 40 days. Abbaspour et al. [34] in *P. vera* cv. Ohadi obtained greater plant length and an increase in stem diameter when inoculating with 1000 spores per plant with the fungus *Rhizophagus irregularis* Becker and Gerdemann (Gec) at concentrations of 0 and 250 mM NaCl for seven days. Chen et al. [27] observed that colonization with *Rhizophagus irregularis* at a dose of 1800 spores per plant and a concentration of 100 mM NaCl promoted greater biomass accumulation in *Z. mays* plantlets.

In this study, the increase in dry matter in the AMF treatments could be due to the fact that *G. intraradices* is an endomycorrhizal fungus. This type of fungus inhabits the cells and tissues of the plant and could cause a relative increase in root dry matter compared to the control treatment, without the presence of colonies in the roots. According to the results obtained, the increase in developmental variables in mycorrhized taro plantlets could be due to better water absorption through the external mycelium and a reduction in the accumulation of Na^+^ and Cl^−^ in the aerial parts of the plants. In addition, the increased growth of plantlets with AMF + NaCl is probably due not only to ion storage in the microcosms, but also to the fungus.

### 2.2. Total Chlorophyll and Carotenoid Content

**Figure 2:**
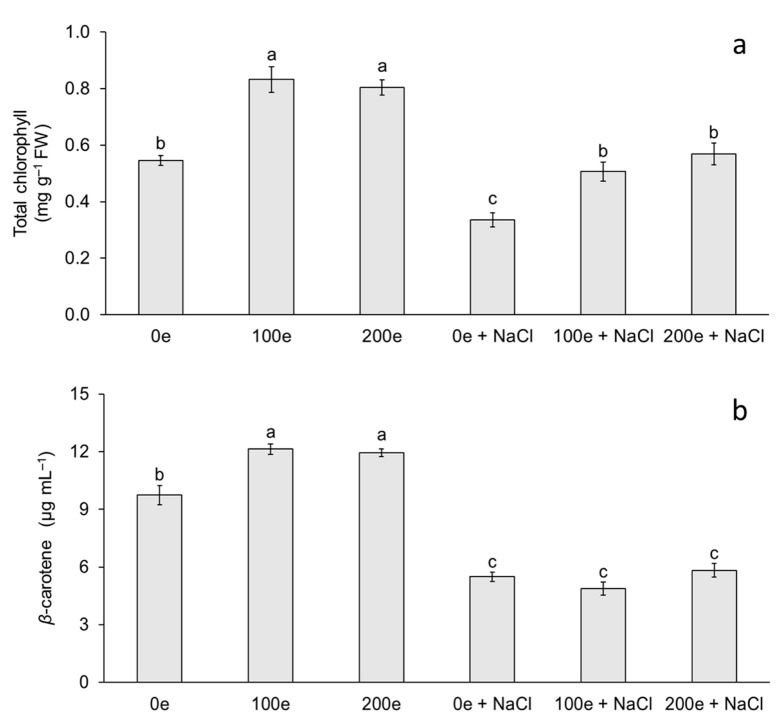
Effect of sodium chloride (NaCl) and arbuscular mycorrhizal fungi (*Glomus intraradices*) on total chlorophyll and *β*-carotene content in taro plantlets (*Colocasia esculenta* L. Schott) during acclimatization stage. (**a**) Total chlorophyll and (**b**) *β*-carotene. Bars represent mean ± standard error. Means with a different letter are significantly different (Tukey, *p* < 0.05). e = spores per plantlet and NaCl is expressed in 100 mM.

The different AMF doses and induced salinity stress had an effect on total chlorophyll and carotenoid content (Figure 2). The highest chlorophyll content was observed in the treatments with 100 and 200 spores per plantlet without salinity stress, whereas the lowest chlorophyll contents were observed when neither mycorrhizae nor salinity stress were applied, and at doses of 100 and 200 spores per plantlet under salinity stress (Figure 2a). For carotenoid content, the highest level was observed in the treatments with 100 and 200 spores per plantlet without salinity stress, whereas the lowest level was observed when mycorrhizae were applied with or without induced salinity stress (Figure 2b).

The results obtained in this study demonstrate the effect of different treatments with mycorrhizae and NaCl on chlorophyll and carotenoid content in taro plantlets. Chlorophyll and carotenoid accumulation in plants inoculated with mycorrhizae has been reported in several species such as tomato (*Lycopersicon esculentum*) [14], sugarcane (*Saccharum* spp.) [36], rice (*Oryza sativa*) [37], and melon (*Cucumis* melo) [29]. Di Martino et al. [14] in *L. esculentum* found that mycorrhizal colonization with *Glomus mosseae* increases the concentration of photosynthetic pigments. Meddich et al. [29] in *C. melo* observed that when using a consortium with *Glomus* sp. at a dose of 44 spores per plant, photosynthetic pigments increased significantly. In this study, total chlorophyll content increased when plantlets were inoculated with mycorrhizae (with or without NaCl); however, plants that were treated with mycorrhizae + NaCl showed an increase in chlorophyll content compared to NaCl treatment without mycorrhizae. Chlorophyll and carotenoid accumulation in plants inoculated with mycorrhizae under salinity stress has been reported in licorice (*Glycyrrhiza glabra*) [32], wheat (*Triticum aestivum*) [33], Russian olive (*Elaeagnus angustifolia*) [38], Saudi pearl millet (*Pennisetum spicatum*) [35], and eucalyptus (*Eucalyptus camaldulensis*) [22]. Amanifar et al. [32] in *G. glabra*, using *Funneliformis mosseae* at 840 spores per plant, found that the relative leaf chlorophyll index increased significantly in salinity stress at a concentration of 40 mM NaCl for 40 days. Liang et al. [38] observed in *E. angustifolia* that total chlorophyll content increased when using *Rhizophagus irregularis* at a dose of 1500 spores per plant under salinity stress with 300 mM NaCl for 21 days. Klinsukon et al. [22] in *E. camaldulensis* observed that total chlorophyll content increased when using a consortium of mycorrhizae (*Glomus* sp., *Gigaspora albida* and *Gigaspora decipiens*) at a dose of 960 spores per plant under salinity stress at a concentration of 100 mM NaCl for 90 days. Variation in chlorophyll content is an important factor that indirectly determines the photosynthetic capacity of plants [39]. A low photosynthetic capacity can disrupt carbon stabilization and eventually decrease the development of plants under stress [39,40]. Increased chlorophyll content in plantlets under salinity stress and with mycorrhizae could be a mechanism to maintain photosynthetic metabolism associated with salinity tolerance. Hashem et al. [41] and Bouskout et al. [42] note that mycorrhizae generate greater availability of N and cofactors involved in chlorophyll synthesis such as Mn, Mg, and Fe. The latter are essential components of the electron carriers in the photosynthetic apparatus, improve stomatal conductance, and consequently increase CO_2_ assimilation. 

In relation to carotenoids, taro plantlets showed a decrease at different doses of mycorrhizae with NaCl (with or without mycorrhizae), with carotenoid content being higher when plantlets were treated with mycorrhizae without NaCl. Ye et al. [28] observed in *C. lanatus* that carotenoid content increased when using *Funneliformis mosseae* at a dose of 700 spores per plantlet and a period of salinity stress with 60 mM NaCl for 6 days. Ndiate et al. [26] observed in *T. aestivum* that carotenoid content increased when using *Glomus mosseae* at a dose of 160 spores per plant in combination with a biochar amendment during a period of salinity stress with 50–150 mM NaCl for 90 days. Therefore, the increase in these antioxidant compounds could decrease the photodegradation and photoinhibition of these photosynthetic pigments by acting as a salinity stress tolerance mechanism [42]. According to Sun et al. [43], carotenoids function as light scavengers for photosynthesis, absorbing light energy and transferring it to chlorophylls in an absorption range of 450–550 nm. Carotenoid levels could be explained by several factors: changes in enzymatic activities, damaged photosynthetic apparatus, inhibition of the electron transport chain, and changes in gene expression involved in the biosynthetic pathway [44]. In this study, the reduction in carotenoid content was probably due to a decrease in their biosynthesis and/or a degradation caused by NaCl-induced salinity stress.

### 2.3. Protein, Proline, and Glycine-Betaine Content

**Figure 3:**
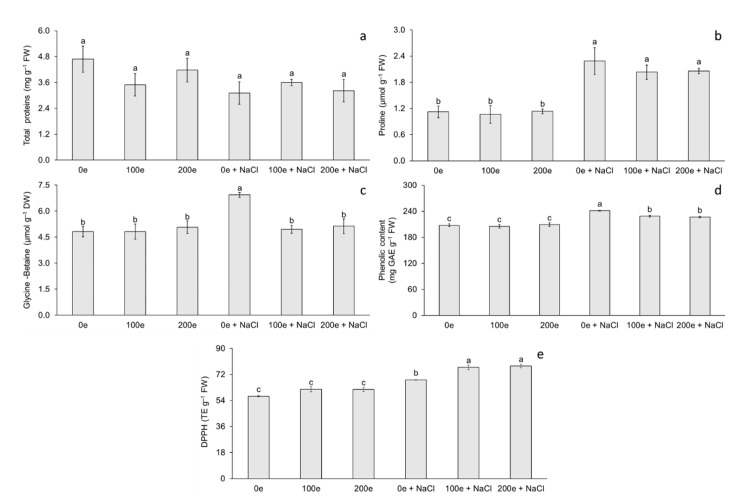
Effect of sodium chloride (NaCl) and arbuscular mycorrhizal fungi (*Glomus intraradices*) on total proteins, proline, and glycine-betaine content in taro plantlets (*Colocasia esculenta* L. Schott) during acclimatization stage. (**a**) Total proteins, (**b**) proline, (**c**) glycine-betaine, (**d**) phenolic content expressed in GAE (milligrams of gallic acid equivalents per g of fresh weight) and (**e**) antioxidant capacity expressed in DPPH (trolox equivalents per g of fresh weight). Bars represent mean ± standard error. Means with a different letter are significantly different (Tukey, *p* < 0.05). e = spores per plantlet and NaCl is expressed in 100 mM.

The different AMF doses and induced salinity stress had no effect on total protein (TP) content; however, differences were observed for proline (Pr) and glycine-betaine (GB) content (Figure 3). The highest proline content was observed when mycorrhizae were applied with or without induced salinity stress, whereas the lowest proline content was observed when salinity stress was not applied, with or without mycorrhizae (Figure 3b). Regarding glycine-betaine, the highest content was observed in plantlets under salinity stress without mycorrhizae, whereas the lowest content was observed in the rest of the treatments (Figure 3c).

The results obtained in this study demonstrate the effect of different doses of mycorrhizae with or without NaCl on Pr and GB content in taro plantlets. Accumulation of TP and/or Pr in plants inoculated with mycorrhizae has been reported in Ashwagandha (*Withania somnifera*) [45], Thymus species (*Thymus daenensis* Celak and *Thymus vulgaris* L.) [46], melon (*Cucumis melo*) [29], and basil (*Ocimum basilicum*) [47]. In *W. somnifera*, Parihar and Bora [45] found that, when using *Glomus mosseae* at 100 spores per plant, the protein content increased significantly. Meddich et al. [29] observed in *C. melo* that when using mycorrhizae with *Glomus* sp. at 44 spores per plant for eight weeks, protein and proline content increased significantly. In this study, TP content did not show significant differences as the plantlets were inoculated with mycorrhizae (with or without NaCl). This was probably due to the specific expression of new proteins under salinity stress in taro; these proteins are not quantifiable by the Bradford [48] method, but rather a qualitative method is required to identify the proteins expressed under salinity stress. However, in other studies the accumulation of TP in plants inoculated with mycorrhizae under salinity stress has been reported in watermelon (*Citrullus lanatus* L.) [28], stevia (*Stevia rebaudiana* B.) [49], eucalyptus (*Eucalyptus camaldulensis*) [22], Saudi pearl millet (*Pennisetum spicatum*) [35], and pistachio (*Pistacia vera* L. cv. Ohadi) [34]. Janah et al. [49] in *S. rebaudiana*, when using a mycorrhizal consortium at 125 spores per plant and at a NaCl concentration of 80 mM for 2 months, found that the protein content increased significantly.

In this study, taro plantlets showed an increase in Pr and GB content when plantlets were treated with NaCl with or without AMF. This effect confirms that Pr and GB are biochemical indicators contributing to salinity stress tolerance mechanisms in taro. Ye et al. [28] observed in *C. lanatus* that Pr content increased when using *Funneliformis mosseae* at a dose of 700 spores per plantlet and a period of salinity stress with 60 mM NaCl for 6 days. Furthermore, [35] found that, in *P. spicatum*, when using *Glomus mosseae* at 2400 spores per plant and at a NaCl concentration of 60 mM for 2 months, the Pr content increased significantly. The Pr is used as a biomarker of osmotic stress; this compatible osmolyte maintains cell turgor, acts as a chaperone protein, and protects cells against free radical damage [50,51].

To date, no GB accumulation has been reported in taro plantlets under salinity stress conditions during the acclimatization stage and inoculated with AMF. However, GB accumulation has been reported in other species during salinity stress. Abbaspour et al. [34] found that, in *P. vera*, when inoculating with 1000 spores per plant with *Rhizophagus irregularis* Becker and Gerdemann (Gec) and at an NaCl concentration of 250 mM for seven days, the GB content increased significantly. GB acts as a compatible osmolyte and promotes antioxidant activity [52]. In addition, it can protect the enzymatic activity of Rubisco and the photosystem II complex during photosynthesis [53].

The symbiosis between AMF and plants under salinity stress could have also induced the synthesis of other compatible osmolytes such as polyamines, sugars, and polyols. These have an important role in osmotic adjustment and protect cells from ROS [54].

### 2.4. Soluble Phenols and Antioxidant Capacity

The different AMF doses and induced salinity stress had no effect on phenol content and antioxidant capacity (DPPH) (Figure 3). The highest phenol content was observed in plantlets under salinity stress without mycorrhizae, whereas the lowest phenol content was observed when no salinity stress was applied, with or without mycorrhizae (Figure 3d). Regarding antioxidant capacity, the highest DPPH content was observed at doses of 100 and 200 spores per plantlet under salinity stress, whereas the lowest DPPH content was observed when salinity stress was not applied, with or without mycorrhizae (Figure 3e).

The results obtained in this study demonstrate the effect of different treatments with mycorrhizae and NaCl on the content of soluble phenols and antioxidant capacity in taro plantlets. The accumulation of soluble phenols and antioxidant capacity in plants inoculated with mycorrhizae has been reported in French tamarisk (*Tamarix gallica*) [55], globe artichoke (*Cynara cardunculus* L. cv. scolymus Fiori) [56], prickly pear cactus (*Opuntia ficus-indica*) [57], and lettuce (*Lactuca sativa* L.) [58]. Bencherif et al. [55] in *T. gallica* found that when using a consortium (*Funnneliformis mosseae*, *Septoglomus constrictum*, *Gigaspora gigantea*, *Glomus* sp1., and *Glomus* sp2.) at a dose of 165 spores per plant, total phenolic content increased significantly more in roots than in leaves. Lahbouki et al. [57] observed in *O. ficus-indica* that when using a consortium with seven genera (*Glomus* (32%), *Acaulospora* (23%), *Rhizophagus* (14%), *Scutellospora* (9%), *Diversispora* (9%), *Claroideoglomus* (9%), and *Gigaspora* (4%)) distributed into 22 species at a dose of 344 spores per plant, total phenolic content increased significantly and antioxidant capacity decreased.

In this study, soluble phenol content increased as plantlets were inoculated with mycorrhizae (with or without NaCl); however, plantlets that were treated with mycorrhizae alone did not show a significant increase in soluble phenol content. The increase in soluble phenols in mycorrhizal-inoculated plantlets under salinity stress has been reported in lettuce (*Lactuca sativa* L.) [59], valerian (*Valeriana officinalis* L.) [60], forage grass (*Lasiurus scindicus* Henrard) [50], and Casuarinaceae species (*Casuarina equisetifolia* and *Casuarina obesa*) [61]. Santander et al. [59] in *L. sativa* observed that when using a native consortium with *Funneliformis mosseae* (MN264635), *Claroideoglomus lamellosum* (MN263071), and *Diversispora celata* (MN264508) at a dose of 2000 spores per plant and a concentration of 40 mM NaCl for 60 days, total phenolic content increased significantly. Malik et al. [50] in *L. scindicus* found that when using a native consortium with *Claroideoglomus etunicatum*, *Funneliformis mosseae*, *Gigaspora margarita*, and *Scutellospora calospora* at a dose of 282 spores per plant and a concentration of 100 mM NaCl, total phenolic content increased significantly.

Therefore, the increase in soluble phenol content could be a biochemical indicator of salinity stress tolerance. In addition, under salinity stress, plants synthesize antioxidant enzymes and non-enzymatic antioxidants as tolerance mechanisms [54]. Phenolic compounds are non-enzymatic antioxidants that scavenge free radicals [62]. Phenolic compounds can scavenge reactive oxygen intermediates while preventing the initiation of subsequent oxidative processes [50]. In addition, phenolic compounds are characterized by the availability of phenolic hydrogens as scavengers of hydrogen-donating radicals and consequently an increase in antioxidant capacity for scavenging activity [57]. 

Similarly, to soluble phenol content, taro plantlets showed an increase in the content of antioxidant capacity at different doses of mycorrhizae with NaCl; however, the highest antioxidant capacity content was obtained when plantlets were treated with mycorrhizae. This effect confirms that soluble phenols and antioxidant capacity are biochemical indicators that contribute to salinity stress tolerance mechanisms. Djighaly et al. [61] in *C. equisetifolia* and *C. obesa* observed that when using *Rhizophagus fasciculatus* (Thaxt.) C. Walker and A. Schüßler strain DAOM227130 at a dose of 648 spores per plant and a concentration of 200 mM NaCl for 15 days, total antioxidant capacity increased significantly. The accumulation of phenolic compounds and antioxidant capacity are a mechanism against oxidative stress. In this study, taro plantlets tolerance to NaCl could be related to the increase in soluble phenols and antioxidant capacity under salinity conditions. 

In this study, the application of *Glomus intraradices* under salinity stress had an effect on plantlet development, photosynthetic pigments, Pr and GB, phenolic compounds, and antioxidant capacity in taro plantlets; no effects on PT accumulation were observed with the evaluated technique. However, the fungus–plant symbiosis specifically induces gene expression, activation of aquaporins, and the activity of enzymatic and non-enzymatic antioxidant compounds related to a salinity stress tolerance response [54,63]. In addition, AMF protect host plants against salinity stress through different mechanisms, where fungal structures (hyphae and mycelia) function as extensions of roots to maintain direct uptake of water and nutrients from the extraradical mycelium, improve soil structure, compartmentalization and ion exchange in vacuoles [64], and induce expression of genes coding for Na and K transporters, as well as H^+^ pumps that regulate ion transport in fungal cells [65]. Taro plantlets exposed to salinity stress showed senescent leaves compared to plantlets inoculated with AMF and without NaCl. In general, AMF-inoculated plantlets had better tolerance to salinity stress through the induction of different physiological and biochemical processes to maintain adequate plantlet development.

## 3. Materials and Methods

### 3.1. Plant Material and Micropropagation

For in vitro establishment of taro (*Colocasia esculenta* L. Schott cv. Criolla), 10 cm apices were collected. The apices were washed with water and Axion complete^®^ commercial soap (Mission Hills, S.A. de C.V., San Jose of Iturbide, GT, Mexico), transferred to the laboratory where they were immersed in a solution containing 1 g L^−1^ fungicide (Cupravit, Bayer AG, Leverkusen, NW, DE) and 1 g L^−1^ bactericide (Agrimycin, Pfizer, New York, NY, USA) for 15 min, and then rinsed with tap water. In a laminar flow hood, the apices were reduced to 2 cm and then rinsed for five min in a 15% (*v*/*v*) solution of Cloralex™ commercial chlorine bleach (Industrias Alen, NL, Mexico) (5% a.i.) and with three drops of Tween 20^®^ (Sigma-Aldrich^®^ Chemical Company, Saint Louis, MO, USA) per 100 mL of water for 20 min. Subsequently, they were immersed in 70% ethanol for 1 min and rinsed three times with sterile distilled water. Finally, 1 cm apical meristems were excised with a scalpel and cultured individually in test tubes containing 10 mL of Murashige and Skoog (MS) [66] medium supplemented with 30 g L^−1^ sucrose and 1 mg L^−1^ BAP (6-Bencilaminopurine, Sigma-Aldrich^®^). The culture medium was adjusted to pH 5.8 with 0.1 N NaOH, after which 2.5 g L^−1^ Phytagel™ (Sigma-Aldrich^®^) were added as a gelling agent before being sterilized in an autoclave at 120 °C and 115 kPa for 15 min. The explants were incubated at 24 ± 2 °C, under irradiance of 40 ± 5 μmol m^−2^ s^−1^ and a photoperiod of 16 h light. After one week of culture, the apices were transferred for the multiplication phase to MS medium supplemented with 3 mg L^−1^ BAP (Sigma-Aldrich^®^). After three subcultures (30 d each), 2 cm shoots were individualized and transferred to MS rooting medium without growth regulators. After 15 days of culture, 5 cm long plantlets were rinsed with tap water and taken to the greenhouse for acclimatization.

### 3.2. Mycorrhizal Fungi Inoculation and Culture Conditions

Inoculation with mycorrhizae was performed under ex vitro greenhouse conditions using ex vitro plantlets with a length of 5 cm and mycorrhizae of the species *Glomus intraradices* (Biofertilizante INIFAP^®^, Chiapas, Mexico). The plant–fungus inoculation was carried out in 32-cavity polypropylene trays with a substrate made up of compost, peat moss, and agrolite (2:1:1 *v*/*v*). The substrate was sterilized in the autoclave for 30 min at 120 °C and 115 kPa. Different doses (0, 100, and 200 spores per plantlet) of *G. intraradices* were evaluated. Taro plantlets were covered with a translucent polyvinyl chloride dome to control humidity conditions. The inoculated plantlets were kept under greenhouse conditions with 60% shade at 30 ± 2 °C, relative humidity of 60 ± 10%, and natural light at an irradiance of 80 ± 10 μmol m^−2^ s^−1^ for 30 days. In a second phase, the dome was removed from the plantlets which were then kept at a temperature of 35 ± 2 °C, relative humidity of 30%, and natural light at an irradiance of 150 ± 10 μmol m^−2^ s^−1^ for 30 d. Throughout the experiment the plantlets were placed above a dome containing 3 L of osmosis water for two months. After 60 d of acclimatization, the plantlets were exposed to salinity stress with 3 L of 100 mM NaCl solution (Fermont, NL, Mexico) for 10 d. The salt concentrations were determined based on a previous study by [31] in *C. esculenta*, which found a maximum survivable concentration of 100 mM NaCl. After the salinity stress time, the colonization percentage, plantlet length, number of leaves, percentage of senescent leaves, roots per plantlet, root length, fresh weight, dry weight, and dry matter percentage were evaluated. In addition, the content of chlorophyll, carotenoids, proteins, proline, glycine-betaine, soluble phenols, and antioxidant capacity were determined.

### 3.3. Mycorrhizal Colonization

To visualize the effect of the different AMF doses on mycorrhizal colonization, segments of the roots were obtained and fixed in 4% paraformaldehyde, and then incubated for 48 h at room temperature. Root segments were washed three times with distilled water and then incubated in 10% KOH for 15 min at 120 °C. An alkaline hydrogen peroxide solution was added and incubated for 20 min at room temperature, after which 0.05% methylene blue (Sigma-Aldrich^®^) was added and incubated for 24 h at room temperature. The samples were observed under a microscope (M5LCD Velab, Co., Pharr, TX, USA) using a 40× objective. The mycorrhizal colonization percentages were determined using the following formula: Percentage of root colonization (%) = No. of infected segments/No. of examined root segments × 100.

### 3.4. Total Chlorophyll and Carotenoid Content

Total chlorophyll content was determined using the methodology proposed by [67]. A total of 250 mg of fresh leaf tissue were taken and macerated in a mortar with 80% acetone and allowed to stand at −4 °C for 24 h in 2.5 mL of 80% acetone. Subsequently, the mixture was filtered using No. 41 filter paper and adjusted to a final volume of 6.25 mL with 80% acetone. Finally, it was read at an absorbance of 663 and 645 nm for chlorophyll a and b, respectively. Readings were made using a spectrophotometer (Thermo Scientific Genesys 10S; Madison, WI, USA). Carotenoid (beta-carotene) content was determined according to the method described by [68] and quantification was done using the following formula:

C = A_450_ × M × 1000/ε × δ

where:

A = absorption determined at 450 nm

M = *β*-carotene molecular mass (537 g^−1^ mol)

ε = molar extinction coefficient of *β*-carotene in acetone (140,663 L^−1^ mol cm).

δ = optical path (cm).

### 3.5. Total Protein (TP) Estimation

The TP estimation was carried out by the method proposed by [48]. Ten milligrams of dry plant material were taken and macerated in a mortar in cold acetone. The macerated tissue was vacuum filtered until obtaining acetone powder. Next, 1.25 mL of 0.1 M tris-HCl pH 7.1 buffer was added. Subsequently, the solution was centrifuged at 3100× *g* for 20 min at 4 °C. Finally, 5 mL of Bradford solution were added to a 0.1 mL sample of the supernatant and read at an absorbance of 595 nm in a spectrophotometer (Thermo Scientific Genesys 10S, Madison, WI, USA). The values were interpolated in the calibration curve made with bovine albumin (Sigma-Aldrich^®^).

### 3.6. Proline (Pr) Determination

Pr was estimated according to the colorimetric method described by [69]. First, 250 mg of fresh leaf tissue were homogenized with 5 mL of 3% sulfosalicylic acid. The resulting paste was sieved through Whatman’s No. 2 filter paper and a 1 mL aliquot was taken, to which 1 mL of glacial acetic acid and 1 mL of ninhydrin were added. This mixture was left to incubate in a thermoregulated bath for 1 h at 100 °C. The tubes were removed and rapidly chilled on ice. Two mL of toluene were added and mixed for 30 s. The chromophore containing the toluene was separated to measure absorbance. The absorbance was read at 520 nm in a spectrophotometer (Thermo Scientific Genesys 10S, Madison, WI, USA) and the values were interpolated in the calibration curve made with L-proline standard (Sigma-Aldrich^®^).

### 3.7. Glycine-Betaine (GB) Determination

GB was determined using the colorimetric method proposed by [70]. First, 250 mg of dry macerated plant tissue were taken, to which 10 mL of deionized water were added and left to incubate for 24 h. An aliquot of 0.5 mL diluted at a 1:1 ratio with 2 N H_2_SO_4_ was taken, after which 0.1 mL of KI-I_2_ was added. The samples were mixed and left under refrigeration at 0–4 °C for 16 h. They were then centrifuged at 3100× *g* for 15 min at 0 °C and placed on ice for 1 h. Finally, the supernatant was separated and 4.5 mL of 1,2-Dichloroethane were added and left at room temperature for 2 h. The chromophore was separated to measure absorbance at 365 nm in the spectrophotometer (Thermo Scientific Genesys 10S, Madison, WI, USA). The values obtained were interpolated in the calibration curve made with glycine-betaine standard (Sigma-Aldrich^®^).

### 3.8. Determination of Soluble Phenols and Antioxidant Capacity

Phenolic content was determined according to [71]. First, 250 mg fresh weight of plant tissue were taken and macerated in a mortar; the extraction was performed with 10 mL of methanol: water (80:20). Subsequently, the solution was centrifuged at 3100× *g* for 10 min at 10 °C. Next, 150 µL of the supernatant were taken and 750 µL of 10% Folin-Ciocalteu’s reagent (E. Merck, Darmstadt, Germany) were added; it was homogenized gently, 600 µL of 20% calcium carbonate (Sigma-Aldrich^®^) were added as well, and then it was incubated for 2 h at 26 °C. Finally, the absorbance was measured at 765 nm using distilled water as a blank. Phenolic content was calculated from a gallic acid calibration curve (0–10,000 µg mL^−1^) and expressed as milligrams of gallic acid equivalents (GAE) per g of fresh weight (g FW) of taro plantlets.

The antioxidant capacity was expressed in DPPH (2, 2-Diphenyl-1-picrylhydrazyl). The DPPH was performed by the methodology proposed by [72]. An aliquot of 2900 µL of DPPH and 100 µL of methanolic extract obtained in the phenolic content determination was taken. The mixture was incubated at 26 °C for 1 h and the absorbance was measured at 515 nm. A calibration curve with Trolox (Sigma-Aldrich^®^) was used at different concentrations. Values obtained were interpolated in the calibration curve and expressed as trolox equivalents (TE) per g of fresh weight (g FW) of taro plantlets.

### 3.9. Experimental Design and Statistical Analysis

All experiments were performed in a completely randomized design and were run in triplicate. An analysis of variance was performed followed by Tukey’s range test (*p* < 0.05) using IBM SPSS^®^ statistical software (version 22 for Windows). The percentage data were transformed with the formula Y = arcsine (√ (×/100)), where × is the value of the percentage.

## 4. Conclusions

In this study, since no significant differences were found for the developmental and biomass variables evaluated at doses of 100 and 200 spores per plantlet, it is suggested to apply 100 spores per plantlet to reduce spore handling, product transport, and arbuscular mycorrhizal fungi cost. Biochemical studies of proline, glycine-betaine, soluble phenols, and antioxidant capacity indicate the degree of plant stress and can be used as early selection markers for salinity-tolerant taro cultivars in breeding programs. It is suggested that future studies use a qualitative technique to determine the specific expression of proteins under salinity stress. In addition, reactive oxygen species production and antioxidant enzyme activities could be evaluated in a future study for a better understanding of salinity stress tolerance. Finally, the arbuscular mycorrhizal fungi evaluated in this study induce salinity tolerance in *C. esculenta* plantlets during the acclimatization stage. These plants can be transplanted to the field for further development under saline soil conditions in order to evaluate their tolerance to salinity stress. 

## Figures and Tables

**Table 1:** Effect of sodium chloride (NaCl) and arbuscular mycorrhizal fungi (*Glomus intraradices*) on development of taro (*Colocasia esculenta* L. Schott) during acclimatization stage.

Mycorrhizae (Spores/Plantlet)	NaCl (mM)	Colonization (%)	Plantlet Height (cm)	Number of Leaves	Senescent Leaves (%)	Roots per Plantlet	Root Length (cm)	Fresh Weight (g)	Dry Weight (g)	Dry Matter (%)
0	0	0.00 ± 0.00 c	13.45 ± 0.63 c	2.00 ± 0.07 bc	2.66 ± 0.33 c	10.00 ± 1.15 c	15.24 ± 1.67 bc	7.16 ± 0.38 c	0.94 ± 0.02 c	12.54 ± 0.87 b
100	0	83.33 ± 4.40 a	17.63 ± 0.31 a	3.04 ± 0.09 a	2.00 ± 0.57 c	17.66 ± 0.88 a	24.89 ± 1.22 a	10.19 ± 0.24 b	1.56 ± 0.08 a	14.29 ± 0.61 ab
200	0	85.00 ± 5.00 a	17.65 ± 0.33 a	3.11 ± 0.10 a	2.33 ± 0.33 c	17.00 ± 1.15 a	22.08 ± 1.04 a	10.30 ± 0.35 b	1.58 ± 0.07 a	13.81 ± 0.49 ab
0	100	0.00 ± 0.00 c	11.28 ± 0.54 d	1.40 ± 0.10 c	43.00 ± 3.60 a	9.00 ± 0.57 c	10.29 ± 1.01 c	5.97 ± 0.21 c	1.25 ± 0.02 bc	13.96 ± 0.44 ab
100	100	61.66 ± 4.40 b	15.40 ± 0.23 b	2.80 ± 0.07 a	20.33 ± 1.45 b	14.00 ± 1.00 ab	15.90 ± 0.77 b	11.96 ± 0.17 a	1.64 ± 0.08 a	15.80 ± 0.61 a
200	100	63.33 ± 3.33 b	16.20 ± 0.38 ab	2.86 ± 0.45 a	21.33 ± 3.52 b	14.33 ± 1.20 ab	15.82 ± 1.34 b	11.96 ± 0.30 a	1.68 ± 0.06 a	16.23 ± 0.55 a

Values represent the mean ± SE (standard error). Different letters (a, b or c) in a column are significantly different (Tukey, *p* < 0.05).

## Data Availability

All data in this study can be found in the manuscript.
